# Personal

**Published:** 1910-02

**Authors:** 


### PERSONAL.

Dr. Arthur G. Bennett, of Buffalo, has been appointed visiting and consulting ophthalmologist for the Craig Colony of Epileptics at Sonyea, N. Y.

Dr. Henry H. Bingham, of Buffalo, was elected president of the Board of Councilmen for the year 1910, upon the organisation of the board at the beginning of the New Year. Dr. Bingham's service in the same capacity for the past two years gives him ample equipment for the duties of the chair, which he has occupied with dignity, observing courtesy and fairness toward the members.

Dr. L. Bradley Dorr, of Buffalo, who was elected a member of the Board of Councilmen, last fall, took his seat January 3, 1910, upon the opening of the legislative session for the New Year. Dr. Dorr will make a capable and conscientious councilman.

Dr. Chauncey Pelton Smith, of Buffalo, has established his offices at 69 W. Chippewa Street. His residence remains at 487 Delaware Avenue.

Dr. Carlos F. MacDonald, of New York, will be the guest of honor at a complimentary to be given by a large group of his friends, at the Hotel Astor, Forty-fourth and Forty-fifth streets and Broadway, at 7.30 o'clock, February 2, 1910. The committee includes some of the distinguished citizens, as well as members of the profession. The Journal offers Dr. MacDonald its best wishes on the joyous occasion.

